# Relationship between vitamin D, iron, and hepcidin in premenopausal females, potentially confounded by ethnicity

**DOI:** 10.1007/s00394-023-03240-7

**Published:** 2023-08-29

**Authors:** Anya Greenwood, Pamela Ruth von Hurst, Kathryn Louise Beck, Hajar Mazahery, Kimberley Lim, Claire Evelyn Badenhorst

**Affiliations:** School of Sport, Exercise and Nutrition, Massey University Auckland, Auckland, New Zealand

**Keywords:** Iron regulation, Serum ferritin, Vitamin D status, Serum 25(OH)D, South Asian

## Abstract

**Purpose:**

To investigate the associations between vitamin D, hepcidin, and iron status in premenopausal females of different ethnic cohorts residing in Auckland, New Zealand (NZ).

**Methods:**

A total of 160 females aged 18–45 years participated in a cross-sectional study. Demographics, body composition, serum 25(OH)D, inflammatory markers (C-reactive protein and interleukin-6, IL-6), and iron biomarkers (serum ferritin, haemoglobin, soluble transferrin receptor, and hepcidin) were measured. Comparisons between parametric, non-parametric, and categorical variables were completed by using one-way ANOVA, Kruskal–Wallis, and Chi-squared tests, respectively. ANCOVA was used to compare serum 25(OH)D across iron parameter categories.

**Results:**

Of the 160 participants, 60 were NZ European, 67 were South Asian, and 33 were from the ‘other’ ethnic groups. South Asians had significantly higher body fat percentage (BF%) and IL-6 concentration (38.34% and 1.66 pg·mL^−1^, respectively), compared to NZ Europeans (27.49% and 0.63 pg·mL^−1^, respectively, *p* < 0.001). South Asians had significantly lower 25(OH)D concentrations compared to NZ Europeans (33.59 nmol·L^−1^ vs 74.84 nmol·L^−1^,* p* < 0.001). In NZ Europeans, higher 25(OH)D concentration was seen in those with lower (≤ 3.5 nM) hepcidin concentration, *p* = 0.0046. In South Asians, higher 25(OH)D concentration was seen in those with higher (> 3.5 nM) hepcidin concentrations, *p* = 0.038. There were no associations between serum 25(OH)D and serum ferritin.

**Conclusion:**

Within South Asian women, an unexpected positive relationship between 25(OH)D and hepcidin concentration was observed which may be due to significantly higher IL-6 concentrations, BF%, and lower 25(OH)D concentrations. Future research is required to confirm these observations in this ethnic cohort.

## Introduction

Globally iron deficiency anaemia (IDA) impacted approximately 29% of non-pregnant females (aged 12–50 years) in 2011 [1]. High rates of iron deficiency (ID) have been observed in New Zealand (NZ), particularly in the South Asian population, with 55.8% of premenopausal females classed as iron deficient (serum ferritin (SF) < 30 ug·L^−1^, haemoglobin (Hb) < 120 g·L^−1^ or ≥ 120 g·L^−1^) [2]. Iron supports oxygen transport and energy production and therefore can result in reduced physical performance, cognitive function, health, and wellbeing of the individual presenting with ID [3].

To ensure iron levels are maintained in a normal and homeostatic range, hepcidin, a peptide hormone encoded by the *HAMP* gene, regulates the expression of the iron export protein ferroportin on hepatocytes, macrophages, and enterocytes [4]. When hepcidin concentration is increased, degradation and internalisation of ferroportin channels occur, resulting in reduced movement and recycling of iron within systemic circulation [4]. Hepcidin production is primarily stimulated by iron status, specifically high iron levels [5]. However, inflammation has been identified as a secondary factor that stimulates hepcidin production, primarily the inflammatory cytokine, interleukin-6 (IL-6) [6]. Individuals presenting in an inflammatory state will have increased IL-6 and subsequently elevated hepcidin activity which has been shown to result in reduced iron absorption and recycling [6]. Increased inflammation and IL-6 levels are associated with obesity, defined as body mass index (BMI) ≥ 30 kg·m^2^, and increased body fat % (BF%) [7, 8]. Specifically, within individuals with an increased ratio of android fat/total body fat, increased inflammatory levels (typically assessed by the presence of C-reactive protein (CRP) and IL-6) have been observed and subsequently were associated with increased serum hepcidin concentration and impaired iron homeostasis and increased risk of iron deficiency [9].

Conversely, hepcidin appears to be suppressed when iron stores are low, during hypoxia when there is an increased erythropoietic demand [4], and by increased serum 25-hydroxyvitamin D (25(OH)D) concentrations suppressing *HAMP* mRNA expression [10]. Individuals with decreased 25(OH)D concentrations are reported in previous research to be at an increased risk of developing ID [11, 12]. In addition, vitamin D_2_ and D_3_ supplementation has been observed to decrease serum hepcidin concentration in supplemental studies [10, 13]. However, the association between the two micronutrients taking into consideration the impact of the regulating hormone, hepcidin, and lifestyle factors that could affect both iron and vitamin D status has not yet been investigated in at-risk population groups within New Zealand.

In 2008/09, 27.1% of adults over the age of 15 years residing in NZ had insufficient vitamin D levels (25(OH)D, 25–49 nmol L^–1^) and 4.9% were vitamin D deficient (25(OH)D < 25 nmol L^−1^) [14]. Despite vitamin D being found in some foods, the impact of dietary intake on vitamin D status is minimal [15], with the main source of vitamin D acquired from the skin’s exposure to ultraviolet β (UVβ) radiation from the sun. Similar to hepcidin, body composition should be considered when assessing an individual’s vitamin D status. Vitamin D is a fat-soluble vitamin; therefore, increased adipose tissue results in increased sequestration of 25(OH)D, resulting in decreased circulating 25(OH)D concentrations [16]. Increased BF% has been identified as a contributing factor to the decreased response to oral vitamin D supplementation and decreased 25(OH)D concentrations [17]. The prevalence of obesity in NZ is high, with 30.9% of NZ adults meeting the classification of obesity in 2019/2020 [18]. Therefore, when considering both iron and vitamin D status in NZ, body composition may be considered as a key cofactor.

Between 2006 and 2018, the total Asian population of NZ nearly doubled in size, with 707,598 permanently residing in NZ in 2018 [19]. Female South Asians are reported to have a higher prevalence of both micronutrient deficiencies, with previous research suggesting that the odds of South Asians having ID are 1.68 times more than NZ Europeans [20]. In addition, South Asians are frequently reported to have lower 25(OH)D (37.0 vs 57.9 nmol L^−1^) compared to NZ Europeans [21]. Therefore, this study aims to investigate the association between vitamin D status, hepcidin, and iron status, stratified according to ethnicity in premenopausal females living in Auckland, NZ.

## Methodology

This was a secondary analysis of data from a cross-sectional study in three ethnic cohorts of pre-menopausal females living in Auckland, NZ. Data collection commenced in July 2018 and concluded in July 2019.

### Participants and recruitment

Participants were recruited through posters, flyers, email contacts, newspaper articles, advertisements, social media, and community groups. Screening to ensure inclusion criteria were met was performed via a questionnaire prior to participants taking part in the study.

Inclusion criteria were healthy premenopausal females, aged between 18 and 45 years (participants over the age of 45 were included if an active menstrual cycle was confirmed) and of South Asian, NZ European, or ‘other’ ethnicity living in Auckland, NZ. Volunteers were excluded if they were: pregnant or breastfeeding, had been pregnant in the last year, had a chronic health condition that may impact iron status (e.g. menorrhagia, coeliac disease, or kidney disease), donated or transfused blood in the last 6 months, or had been consuming iron supplements (> 20 mg elemental iron), 3–4 times a week in the 3 months prior to participation.

Ethics approval was obtained from Massey University Human Ethics Committee: Southern A (18/12). Prior to data collection, the participants were provided with an information sheet that informed them of the study procedures. Written informed consent was obtained from all individual participants included in the study.

The following formula was used to calculate the sample size: *N* = [*Z*2 *p*(1–*p*)]/*d*2. A 95% level of confidence, with a corresponding *Z* score of 1.69 (*Z*), 5% precision (*D*), and based on an estimation of 12.1% (*p*) prevalence of ID in the population of interest [22]. A sample size of 162 participants was determined to be adequate.

### Body composition measurements

Height (to the nearest cm) was measured using a stadiometer by a trained researcher following a standardised procedure. Height was recorded three times and then averaged. Body composition was measured using bioelectrical impedance analysis (InBody 230). The data recorded included total body weight and BF%. BMI was calculated using participant’s weight (kg) divided by their height (m) squared. BMI classifications were based on previous population data from within New Zealand [23], while the BF% threshold was set as the median of the study population.

### Blood sample analysis

Haemoglobin was measured using a HemoCue Hb 201+ system via a single finger prick blood sample [24], to ensure whole blood sample analysis of haemoglobin at the time of data collection. A trained phlebotomist collected a venous blood sample. Once all blood samples were collected, serum was sent to LabPlus Auckland and Canterbury Health Laboratory for markers of iron status (serum ferritin (SF) and soluble transferrin receptor (STfR)), inflammation (CRP), and 25(OH)D. For 25(OH)D analysis, an electrochemiluminescence immunoassay using Roche COBAS^®^ e411 system (Roche Diagnostics, Indianapolis, IN, USA), CV of 10.3% was used. Serum interleukin-6 (RD Systems Human IL-6 Immunoassay high sensitivity ELISA, D6050) and hepcidin (RD System Human Hepcidin Immunoassay ELISA, HDP250) were analysed via commercially available ELISA kits at the Human Nutrition Research Unit at Massey University, Auckland. The coefficient of variation for IL-6 and hepcidin-25 was < 5%. SF < 30 µg·L^−1^ was used as the cut-off value as this is an indicator of stage one ID and is a more sensitive marker for identifying ID [23]. A hepcidin stratification cut-off point of ≤ 3.5 nM and > 3.5 nM was used based on the median hepcidin concentration of our cohort. Hepcidin concentration ≤ 3.09 nM has been identified to be the threshold for increased iron absorption rates [24]. A correction factor was used to adjust the SF values of participants who presented with CRP concentration ≥ 5 mg L^−1^ [25]. 25(OH)D reference ranges were based on those of the New Zealand Ministry of Health [26] and several other international organisations [27].

### Questionnaires

Questionnaires were completed online using the software Qualtrics on iPads or on a personal mobile devices of the participants. Information obtained included demographic (e.g. age, ethnicity) and medical history (e.g. medications, previous chronic disease, previous iron diagnosis, use of iron supplements). Participants were asked about their menstrual blood loss and blood donation as both are known to affect iron status in females [28]. Details of the full methods and use of validated questionnaires have previously been published [20].

### Data handling and statistical analysis

Statistical analysis was carried out using IBM SPSS statistics version 27 (Armonk, NY, USA). All data were tested for normality using the Kolmogorov–Smirnov and Shapiro–Wilk test. Non-normally distributed data were log-transformed to obtain normality. Normally distributed data are reported as mean ± standard deviation (SD) or geometric mean (95% confidence interval). Data that were not normality distributed are reported as median (25th, 75th percentiles). Categorical data are reported as count and percentage.

Comparison between participants and ethnic groups for parametric data was conducted using one-way analysis of variance (ANOVA), followed by Tukey’s post hoc tests to identify where the differences occurred. Comparison between nonparametric data was conducted through Kruskal–Wallis test. For identified significant differences, Mann–Whitney *U* was conducted using NZ European as a reference category and a Bonferroni correction (0.05/2 = 0.025). Pearson’s Chi-squared test was used to compare categorical variables, with the expected cell count for each cell ≥ 5 and all variables considered to be independent. To meet the expected cell count for Chi-square test, BMI categories were collapsed into two groups.

Analysis of covariance (ANCOVA) was used to compare serum 25(OH)D across different iron parameter categories (hepcidin, SF, Hb, and iron status (sufficient: SF ≥ 30ug·L^−1^ and Hb ≥ 120 g·L^−1^ and insufficient: SF < 30 ug·L-1 and Hb < 120 g·L^−1^ or ≥ 120 g·L^−1^)) while adjusting for possible covariates where appropriate (IL-6, BF%, ferritin, and season of enrolment). The interaction effect of covariates on 25(OH)D and iron parameters was examined. Because we found a trend for an interaction effect of ethnicity on the relationship between 25(OH)D and hepcidin (*p* = 0.06), all analyses were stratified by ethnicity, and the results are reported for each ethnic group separately.

## Results

### Participant characteristics

Of the 170 females recruited, 10 participants were excluded: 5 had no blood samples, one had a 25(OH)D concentration of 264 nmol·L^−1^, and 4 blood samples were unmatched to the questionnaire. Out of the 160 participants included in the final analysis, 15 had donated blood, with 6 of those participants donating blood in the last year. Of those included in the final analysis, 51 participants (32%) reported irregular menstrual bleeds (regular menstrual bleeds were those occurring every 24–34 days).

From the self-reported data, three ethnic groups were identified for this study: 67 (42%) South Asian, 60 (38%) NZ European, and 33 (21%) ‘other’ ethnic group. The ‘other’ ethnic group included Middle Eastern, other Asian, Māori, and Pacific. Characteristics of participants stratified by ethnicity are presented in Table 1. South Asians were significantly older compared to NZ Europeans (*p* < 0.001). South Asians had higher BMI and BF % compared to NZ Europeans (Table 1). Nutrition habits of women were not analysed in this study but are reported in Lim et al. [20]. Blood samples were collected across the year, with the majority of South Asians (56%) enrolled during summer/autumn, and most of the NZ Europeans (52%) enrolled during winter/spring.

**Table 1:** Participants’ characteristics for the total population and stratified by ethnicity

Variables	Total *n* = 160	NZ European *n* = 60	South Asian *n* = 67	Other^a^ *n* = 33	*p* value^b^
Age (years), median (25th, 75th centile)	26 (22, 36)	23 (20, 30)	35 (27, 40)^c^	23 (21,28)	< 0.001
Anthropometrics and body composition					
BMI (kg/m^2^), geometric mean (95% CI)	24 (24, 25)	23 (22, 24)	26 (25, 27) ^c^	24 (22, 26)	0.001
BMI, *n* (%)					
< 25 kg/m^2^	95 (59)	44 (46)	30 (32)	21 (22)	0.004
≥ 25 kg/m^2^	65 (41)	16 (25)	37 (57)	12 (19)	
BF%, mean ± SD	33 ± 9.8	27 ± 7.7	38 ± 8.8^c^	31 ± 9.4	< 0.001
BF% categories ^d^, *n* (%)					
< 32%	77 (48)	44 (57)	14 (18)	19 (25)	< 0.001
≥ 32%	83 (52)	16 (19)	53 (64)	14 (17)	

*BMI* body mass index; *BF%* body fat percentage; *CI* confidence intervals; *P/w* number/frequency of servings per week

^a^‘Other’ ethnic groups include Middle Eastern, other Asian, Māori, and Pacific

^b^ANOVA and Kruskal–Wallis tests were performed for normally and non-normally distributed data, respectively. Ethnic groups were compared against NZ European (reference category), and the analyses were adjusted for multiple comparisons (Bonferroni correction; 0.05/2 = 0.025). Pearson’s Chi-squared test was used to compare categorical data

^c^Significantly different to NZ Europeans

^d^Based on the median of study population

Table 2 summarises the biomarkers for the total sample (*n* = 160) and is stratified by ethnicity. The median 25(OH)D for the total population was 54 (33, 73) nmol·L^−1^, with 61% of NZ Europeans presenting with 25(OH)D levels > 50 nmol·L^−1^. Conversely, 78% of South Asians presented with 25(OH)D levels < 25 nmol·L^−1^, and 31% of the ‘other’ ethnicities presented with 25(OH)D levels between 25 and 50 nmol L^−1^. Of the total sample, 89 (56%) of participants had insufficient iron stores (SF < 30 µg L^−1^ and Hb < 120 g·L^−1^ or ≥ 120 g·L^−1^). South Asians had lower 25(OH)D (*p* < 0.001) and Hb (*p* < 0.001) concentrations and significantly higher IL-6 (*p* < 0.001) concentrations when compared to the NZ European cohort. Furthermore, NZ Europeans had significantly higher 25(OH)D (*p* < 0.001) concentrations compared to ‘other’ ethnic groups.

**Table 2:** Iron, vitamin D, hepcidin, and inflammatory biomarkers for the total population and stratified according to ethnicity

Biomarker	Total	NZ European *N* = 60	South Asian *N* = 67	Others^a^ *N* = 33	*p* value^b^
Serum 25(OH)D (nmol L^−1^), Geometric mean (95% Cl)	48 (44, 53)	75 (69, 81)	34 (29, 38)^e^	44 (37, 52)^e^	< 0.001
Vitamin D status, *n* (%)					
≥ 50 nmol·L^−1^	94 (59)	57 (61)	22 (23)	15 (16)	< 0.001
26–49 nmol·L^−1^	39 (24)	3 (8)	24 (62)	12 (31)	
≤ 25 nmol·L^−1^	27 (17)	0	21 (78)	6 (22)	
> 3.5 nM	78 (50)	30 (39)	32 (41)	16 (21)	0.996
≤ 3.5 nM	79 (50)	30 (38)	33 (42)	16 (20)	
Haemoglobin (g·L^−1^), Mean (SD)	131, 13	137, 11	124, 13^f^	133, 10	< 0.001
Serum ferritin (ug·L^−1^), Geometric mean (95% Cl)	24 (21, 28)	25 (21, 30)	20 (16, 25)	31 (22, 45)	0.149
Hepcidin (nM), Geometric mean (95% Cl)	4.3 (3.8, 4.9)	4.2 (3.4, 5.1)	4.4 (3.6, 5.5)	4.6 (3.5, 6.0)	0.947
Soluble transferrin receptor (mg·L^−1^), median (25th, 75th percentile)	2.9 (2.5, 3.5)	2.8 (2.4, 3.4)	3.0 (2.6, 3.7)	2.6 (2.3, 3.3)	0.077
Iron status^f^, *n* (%)					0.239
Sufficient	71 (44)	28 (39)	25 (35)	18 (25)	
Insufficient	89 (56)	32 (36)	42 (47)	15 (17)	
Interleukin-6 (pg·mL^−1^), median (25th, 75th percentile)	1.1 (0.6, 1.7)	0.6 (0.5, 1.0)	1.7 (1.3, 2.5)^e^	0.8 (0.6, 1.1)	< 0.001
Interleukin-6 categories^d^, *n* (%)					
> 1.1	71 (45)	11 (16)	53 (75)	7 (10)	< 0.001
≤ 1.1	86 (55)	49 (57)	12 (14)	25 (29)	

^a^Other ethnic groups include Middle Eastern, other Asian, Māori, and Pacific

^b^ANOVA and Kruskal–Wallis tests were performed for normally and non-normally distributed data, respectively. Ethnic groups were compared against NZ European (reference category), and the analyses were adjusted for multiple comparisons (Bonferroni correction; 0.05/2 = 0.025). Pearson’s Chi-squared test was used to compare categorical data

^c^Hepcidin stratification cut-off point of ≤ 3.5 nM and > 3.5 nM was used based on the median hepcidin concentration of our cohort. Hepcidin concentration ≤ 3.09 nM was identified to be the cut-off point for increased iron absorption rates [24]

^d^Based on median

^e^Significantly different to NZ Europeans

^f^Sufficient: Serum ferritin ≥ 30ug·L^−1^ and Hb ≥ 120 g·L^−1^. Insufficient; Serum ferritin < 30ug·L-1 (Hb < 120 g·L^−1^ or ≥ 120 g·L^−1^)

Table 3 summarises the association of 25(OH)D with hepcidin, SF, haemoglobin, and iron status stratified by ethnicity. There was a trend for an interaction effect of ethnicity on the association of 25(OH)D with hepcidin (*p* = 0.06). In NZ Europeans, higher 25(OH)D concentrations (80 (71, 89) nmol L^−1^) were seen in those with lower (≤ 3.5 nM) hepcidin concentrations, while NZ Europeans with lower 25(OH)D concentrations (70 (63, 78) nmol·L^−1^) tended to have higher hepcidin concentrations (> 3.5 nM) *p* = 0.046. Conversely in South Asians, higher 25(OH)D concentrations (38 (31, 47) nmol L^−1^) was seen in those with higher (> 3.5 nM) hepcidin concentrations, compared to South Asians presenting with lower (≤ 3.5) hepcidin concentrations (29 (25, 35) nmol·L^−1^), *p* = 0.038. No association was observed between 25(OH)D and hepcidin in the ‘other’ ethnic group and between 25(OH)D and other iron parameters in all ethnic groups (*p* > 0.05).

**Table 3:** Associations between serum 25(OH)D, hepcidin, serum ferritin, haemoglobin, and iron status, stratified according to ethnicity

	Serum 25(OH)D, nmol·L^−1^			
	Total	NZ European	South Asian	Others^f^
Hepcidin (nM)				
≤ 3.5	47 (40, 53)	80 (71, 89)	29 (25, 35)	44 (33, 58)
> 3.5	50 (44, 57)	70 (63, 78)	38 (31, 47)	45 (35, 58)
* p* value^e^	0.380	0.046	0.038	0.849
Serum ferritin (µg·L^−1^)				
< 30	46 (41, 52)	74 (66, 84)	34 (29, 40)	40 (31, 53)
≥ 30	50 (44, 58)	75 (69, 83)	33 (25, 43)	49 (38, 61)
* p* value^e^	0.498	0.927	0.799	0.173
Haemoglobin (g·L^−1^), median (25th, 75th centile)				
< 120	34 (24, 61)	93 (69, 97)	28 (21, 45)	54 (34, 69)
≥ 120	59 (38, 76)	73 (64, 87)	39 (24, 53)	48 (31, 65)
* p* value^e^	0.066	0.649	0.437	0.535
Iron status^d^ *n* (%)				
Insufficient	54 (30)^b^	75 (66, 85)	33 (29, 39)	40 (30, 53)
Sufficient	58 (27)^b^	75 (68, 82)	34 (26, 44)	48 (38, 60)
* p* value^e^	0.533	0.918	0.864	0.297

^a^Reported as geometric mean (95th centile) unless otherwise stated

^b^Mean (± SD)

^c^Median (25th, 75th percentile)

^d^Sufficient; Serum ferritin ≥ 30ug·L^−1^ and Hb ≥ 120 g·L^−1^. Insufficient: Serum ferritin < 30ug·L-1 (Hb < 120 g·L^−1^ or ≥ 120 g·L^−1^)

^e^ANCOVA was conducted to identify significant differences using *p* value of ≤ 0.05 as significant. Covariates included IL-6, BF%, ferritin, and season of enrolment

^f^Other ethnic groups include Middle Eastern, other Asian, Māori, and Pacific

## Discussion

This research has identified an ethnic-specific association between 25(OH)D and serum hepcidin concentrations, specifically in South Asian and NZ European premenopausal females.

Within this cohort of participants, NZ Europeans who tended to have lower (≤ 3.5 nM) hepcidin concentration had higher (80 nmol·L^−1^) 25(OH)D concentration. Such results would be in alignment with research from vitamin D supplement studies, demonstrating that one week following a single oral dose of vitamin D_3_ in healthy adults (*n* = 28), a 73% decrease in hepcidin was observed [19]. Similarly, Bacchetta et al. [10] observed a decrease in hepcidin concentration by 34%, 24 h following a single oral dose of vitamin D_2_ (100,000 IU). Previous research has provided evidence to suggest that the reduction in serum hepcidin levels is likely due to the direct binding of 1α,25(OH)_2_D_3_ to the vitamin D receptor present on the *HAMP* promoter gene, decreasing *HAMP* mRNA expression [10]. The results in the NZ Europeans would suggest that in the absence of vitamin D supplementation, adequate vitamin D levels concentrations are likely to be associated with low levels of hepcidin. However, since this study did not measure *HAMP* gene expression, it is likely that basal iron status is still the primary determinant of variations in serum hepcidin [5], but that adequate vitamin D levels may support this appropriate homeostatic hepcidin activity. Future research may be required to verify these findings, particularly in the absence of vitamin D supplementation.

Conversely, South Asians in this study who tended to have higher (> 3.5) hepcidin concentrations had higher (38 nmol L^−1^) 25(OH)D concentrations. This finding contradicts the association observed in the NZ Europeans and results reported previously in other cohorts (Caucasian, African American, and Korean) [10, 13]. To our knowledge, this association has not been reported in any of the previously published literature. Within our cohort, the South Asians had significantly higher BF% (38.34%) and BMI (25.88 kg/m^2^) compared to NZ Europeans (27.49% and 22.91 kg/m^2^, respectively). It is possible that the higher BF% could be a contributing factor to the increased IL-6 concentration observed in South Asians. Interleukin-6 is known to be released by adipose tissue and is directly correlated with adiposity [8], in addition to its role as an upregulator of *HAMP* gene transcription. It is worth noting that body composition has been demonstrated to be associated with ethnicity [29, 30]. For the same BMI, Asians are frequently reported to have higher BF% compared to Caucasians, this is particularly prominent in South Asians, Malay, and Chinese [30]. Furthermore, it has been observed that South Asian females have a higher fat to lean mass ratio compared to Australian Aboriginal, Chinese, and European females [29]. Specifically in NZ, Asian Indians were identified to have 8% higher BF% compared to Europeans [31]. Potential factors that could be contributing to the varying body composition in ethnic groups include genetics, environmental, and intra-uterine development [30]. Therefore, it is possible that the higher BF% and IL-6 levels may be contributing factors to the increased hepcidin levels and the positive association with 25(OH)D levels observed in the South Asian participants within this study.

There are additional factors that may have contributed to the contradictory relationship between hepcidin and 25(OH)D in South Asian premenopausal females within the study. Firstly, it was noted that the geometric mean of 25(OH)D in the cohort of South Asians was lower compared to NZ Europeans and ‘other’ ethnic groups. Only 33% of South Asians were identified as having adequate vitamin D levels (≥ 50 nmol·L^−1^). In comparison, 95% of NZ Europeans had adequate 25(OH)D concentration with a geometric mean of 75 nmol L^−1^. In NZ, South Asians have been identified as a high-risk group for vitamin D deficiency [32]. In a cohort of 228 South Asian females, the median 25(OH)D concentration was 28 nmol L^−1^, with only 16% of the cohort presenting with sufficient (≥ 50 nmol·L^−1^) 25(OH)D concentrations [32]. Previous research studies have demonstrated that adequate levels of vitamin D are likely to have anti-inflammatory actions through the downregulation of T helper 1 cells, subsequently reducing the production of pro-inflammatory cytokines, including IL-6 [33]. Furthermore, in vitro research has demonstrated a dose-dependent relationship between 1,25(OH)_2_D_3_, reducing pre-hepcidin cytokines, IL-6, and IL-1β [34]. Therefore, there is the possibility that the lower mean 25(OH)D concentration observed in South Asians may not be sufficient to exert a suppressive effect on IL-6 concentrations or the *HAMP* transcription, and as a result, hepcidin levels may have remained elevated in this ethnic group within this study. However, this proposition requires further investigation with research that will determine if there is a dose-dependent effect of 25(OH)D on IL-6 and hepcidin expression in different ethnic cohorts.

Within this study, no association was identified between 25(OH)D and SF concentration in any ethnic group within this research. Such results would conflict with those noted in Caucasian female athletes and African Americans, where individuals who presented as vitamin D deficient (< 75 nmol L^−1^) were noted as being at a greater risk of iron deficiency [11]. Conversely, the association between vitamin D levels and iron deficiency risk was not noted for the Caucasians within a mixed-gender cohort [12], a result that is similar to the lack of association between vitamin D and SF noted in this current study. The inconsistencies in the association between vitamin D and iron deficiency status are not limited to cross-sectional research studies but have also been noted in vitamin D supplement trials [12]. Due to the inconsistencies in research design, gender, and ethnicity, future research is required to verify if the association between vitamin D and iron deficiency risk is associated with SF or hepcidin.

Finally, the hepcidin cut-off for stratification used in the study (> 3.5 or ≤ 3.5 nM) was based on the median hepcidin concentration of our cohort. However, this threshold has been suggested by recent research as a possible determinant of iron depletion, with previous results demonstrating increased iron absorption rates in females when hepcidin concentrations fell below ≤ 3.09 nM [35]. Within this previous research, SF of 51.1 µg·L^−1^ corresponded to hepcidin levels of 3.09 nM and was subsequently suggested as an initial threshold required for iron deficiency detection in young premenopausal females [35]. However, within the current study, SF means for all ethnic groups was below 51.1 µg·L^−1^, and therefore, all participants may have been at risk of iron depletion and in time iron deficiency, regardless of vitamin D concentrations. Within the NZ Europeans, reduced hepcidin concentration observed in those with higher 25(OH)D concentrations may suggest adequate homeostatic response of hepcidin to the individual’s current iron status. However, in South Asians, despite the reduced SF concentration, hepcidin remained elevated. This may suggest additional confounding factors (e.g. BF%, IL-6, dietary intake) could strongly influence hepcidin concentration when iron stores are reduced, thus impacting iron homeostasis in this cohort.

This study is the first cross-sectional study in healthy premenopausal females that includes measures of serum hepcidin when investigating the associations between vitamin D and iron status. Due to the complementary and then conflicting observation of 25(OH)D and hepcidin concentration in NZ European and South Asians, respectively, further research is needed to increase knowledge on the dose-dependent effect of vitamin D on hepcidin concentrations and iron regulation in these populations. Consideration for this future research is the assessment of vitamin D binding protein, which is known to be ethnically diverse and may contribute to the interaction between vitamin D and hepcidin that was observed in the current study [36]. In addition, researchers may consider conducting research in larger samples of each of the ethnic groups identified in the current study, as we acknowledge that the current power calculation of the study was not ethnic group specific. Finally, to improve the results presented here, researchers may seek to collect data in defined seasons and in age-matched groups to reduce any influence this may have on the iron and vitamin D results.

## Conclusion

Within NZ premenopausal females, ethnic differences in the association between serum hepcidin and 25(OH)D were noted. Results within the NZ Europeans complement that previously reported and demonstrate an inverse relationship between hepcidin and vitamin D levels, a result that may facilitate improved iron regulation when SF levels are low. However, the association between serum hepcidin and vitamin D in South Asians would contradict much of the available research. Within this study, noted confounding factors that may have influenced this result include significantly higher BF% which may have contributed to elevated IL-6 levels in South Asians. Due to the novelty of these research results, future investigations are required to validate the findings reported here in each of the ethnic cohorts.

## Data Availability

Data is stored at Massey University. Access to data for subsequent analysis may only be obtained through enquiry and discretion of Dr Badenhorst.
